# Exploring pharmacological activities and signaling of morphinans substituted in position 6 as potent agonists interacting with the μ opioid receptor

**DOI:** 10.1186/1744-8069-10-48

**Published:** 2014-07-24

**Authors:** Tanila Ben Haddou, Davide Malfacini, Girolamo Calo, Mario D Aceto, Louis S Harris, John R Traynor, Andrew Coop, Helmut Schmidhammer, Mariana Spetea

**Affiliations:** 1 Department of Pharmaceutical Chemistry, Institute of Pharmacy and Center for Molecular Biosciences Innsbruck (CMBI), University of Innsbruck, Innrain 80-82, Innsbruck A-6020, Austria; 2 Department of Medical Sciences, Section of Pharmacology and Italian Institute of Neuroscience, University of Ferrara, Ferrara I-44121, Italy; 3 Department of Pharmacology and Toxicology, School of Medicine, Virginia Commonwealth University, Richmond, VA 23298-0613, USA; 4 Department of Pharmacology, University of Michigan Medical School, 1301 MSRB III, 1150 West Medical Center Drive, Ann Arbor, MI 48109-5632, USA; 5 Department of Pharmaceutical Sciences, University of Maryland, School of Pharmacy, Baltimore, MD 21201, USA

**Keywords:** Opioid receptors, Agonist, Morphine, Oxycodone, Pain, Analgesia, Signaling, G protein, Calcium mobilization

## Abstract

**Background:**

Opioid analgesics are the most effective drugs for the treatment of moderate to severe pain. However, they also produce several adverse effects that can complicate pain management. The μ opioid (MOP) receptor, a G protein-coupled receptor, is recognized as the opioid receptor type which primarily mediates the pharmacological actions of clinically used opioid agonists. The morphinan class of analgesics including morphine and oxycodone are of main importance as therapeutically valuable drugs. Though the natural alkaloid morphine contains a C-6-hydroxyl group and the semisynthetic derivative oxycodone has a 6-carbonyl function, chemical approaches have uncovered that functionalizing position 6 gives rise to a range of diverse activities. Hence, position 6 of *N*-methylmorphinans is one of the most manipulated sites, and is established to play a key role in ligand binding at the MOP receptor, efficacy, signaling, and analgesic potency. We have earlier reported on a chemically innovative modification in oxycodone resulting in novel morphinans with 6-acrylonitrile incorporated substructures.

**Results:**

This study describes *in vitro* and *in vivo* pharmacological activities and signaling of new morphinans substituted in position 6 with acrylonitrile and amido functions as potent agonists and antinociceptive agents interacting with MOP receptors. We show that the presence of a 6-cyano group in *N*-methylmorphinans has a strong influence on the binding to the opioid receptors and post-receptor signaling. One 6-cyano-*N*-methylmorphinan of the series was identified as the highest affinity and most selective MOP agonist, and very potent in stimulating G protein coupling and intracellular calcium release through the MOP receptor. *In vivo*, this MOP agonist showed to be greatly effective against thermal and chemical nociception in mice with marked increased antinociceptive potency than the lead molecule oxycodone.

**Conclusion:**

Development of such novel chemotypes by targeting position 6 provides valuable insights on ligand-receptor interaction and molecular mode of action, and may aid in identification of opioid therapeutics with enhanced analgesic properties and fewer undesirable effects.

## Background

Pain is a physiological integrated part of human life and protects the body from any potentially dangerous thermal, mechanical or chemical injury. This biological process involves different regulation levels (peripheral, spinal, supraspinal), where pain signals are transmitted and modified along the pain pathways, reaching the brain and resulting in pain awareness [1]. Moreover, pain is a complex multidimensional phenomenon, and its highly subjective nature makes it difficult to define and to treat clinically. Nowadays, effective pain management is still a therapeutic priority, with pain being an incapacitating symptom of many medical conditions [2,3].

Among the three opioid receptor types, μ (MOP), δ (DOP) and κ (KOP), the MOP receptor is the main type involved in modulation of pain perception, and it has the most clinical value in pharmacotherapy of pain with opioid analgesics [4]. Similar to the other two receptors, DOP and KOP, the MOP receptor displays the topology characteristics of the rhodopsin family of G protein-coupled receptors (GPCRs) with seven transmembrane loops [5], and is expressed at central and peripheral sites within the pain control circuits. An important milestone in the opioid field, represented by elucidation of the MOP receptor structure, was recently reached [6].

**Figure 1:**
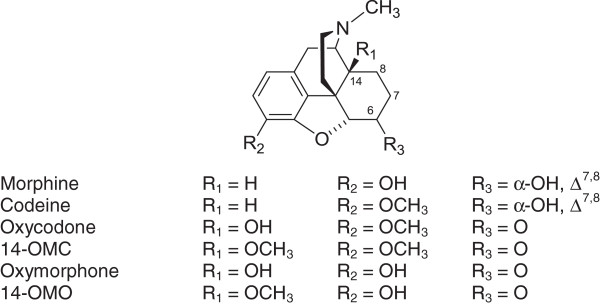
**Structures of morphine, codeine, oxycodone, 14-*O*-methyloxycodone (14-OMC), oxymorphone and 14-*O*-methyloxymorphone (14-OMO).**

Morphine (Figure 1) and all structurally related commonly used opioid analgesics for the treatment of moderate to severe pain are agonists at the MOP receptor [4,7]. Morphine has high effectiveness as an analgesic drug and a long history of clinical use, in spite of its serious side effects, such as constipation, respiratory depression, sedation, nausea and vomiting [8]. Codeine (3-*O*-methylmorphine, Figure 1) is used as analgesic for mild to moderate pain, with 5 to 6 times less potency than morphine [7]. The morphinan oxycodone (Figure 1) is nowadays one of the most frequently used opioid analgesics, with potency comparable to that of morphine [9]. A main problem associated with oxycodone is its high abuse potential. 3-*O*-Demethylation of oxycodone leads to an active metabolite, oxymorphone (Figure 1), a clinically used analgesic, while also representing a valuable scaffold for the development of new generations of ligands interacting with the MOP receptor [10-12]. Over the years, intensive investigations have been directed toward optimization of morphinan-6-ones, and significant developments and the therapeutic potential of the generated molecules or their use as valuable research tools have been reported. Our work in the 6-ketomorphinan class of opioid analgesics has led to the design of 14-alkoxy substituted morphinan-6-ones as MOP agonists that are highly effective antinociceptive agents in various experimental models of pain [10,11]. Replacement of the hydroxyl group in position 14 of oxymorphone with a methoxy group led to 14-*O*-methyloxymorphone (Figure 1), which shows 9 times increased binding affinity at the MOP receptor [13] and is up to 40 times more potent in inducing an antinociceptive effect than oxymorphone in animals [14]. Furthermore, position 6 of *N*-methylmorphinans has been extensively targeted, and found to play a key role in ligand interaction with the MOP receptor, and also analgesic properties. Medicinal chemists have taken the synthetic approach of converting the C-6 carbonyl group into various functionalities, leading to hydrazones, oximes, carbazones and semicarbazone derivatives of *N*-methyl-6-ketomorphinans [15-18], where high antinociceptive potency was combined with reduced unwanted side effects like respiratory depression and gastrointestinal inhibition [19,20]. Zwitterionic molecules with interesting pharmacological profiles were designed through the introduction of 6 amino acid residues in 14-alkoxymorphinans [21-27]. Such MOP receptor agonists induce potent and long-lasting peripherally mediated antinociceptive actions after systemic subcutaneous (s.c.) administration. Recently, we have reported on the development of 14-*O*-methyloxymorphone derivatives with an amino and guanidino group in position 6 [28]. These opioid compounds display high MOP affinity, selectivity and efficacy, and were very active as antinociceptive agents.

**Figure 2:**
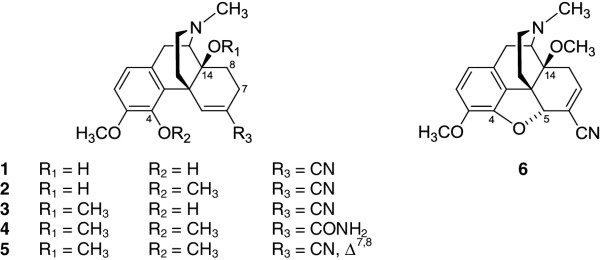
**Structures of *N*-methylmorphinans 1–6.**

Another synthetic strategy to convert the carbonyl group of morphinan-6-ones was described by the replacement of the 6-keto group with an acrylonitrile substitution to provide the *N*-methylmorphinans **1**–**3** (Figure 2) [29,30], without major alterations in the opioid activities *in vitro* and *in vivo* compared to their 6-keto analogues [30]. The resulted compounds exhibited high affinity at the MOP receptor, MOP selectivity and antinociceptive potencies [30]. However, the presence in positions 4 or 14 of a methoxy instead of a hydroxyl group in the 6-cyanomorphinans affects the interaction with opioid receptors, by increasing affinity and selectivity for the MOP receptor, together with augmenting antinociceptive potencies [30]. With the aim of extending structure-activity relationships (SAR) in this class of compounds, further 6-acrylonitrile incorporated *N*-methylmorphinans (**5** and **6**) and the corresponding amido derivative **4** have been synthesized [31] (Figure 2). The present study was undertaken to evaluate their *in vitro* biological properties (opioid receptor binding and functional activities), and *in vivo* behavioral properties (nociception), in comparison to the previously reported analogues **1**–**3**, and the lead molecule oxycodone. While no data have been reported on the effect of 6-cyanomorphinans **1**–**3** on G protein coupling or second messenger signaling, herein we describe the *in vitro* characterization of compounds **1**–**6** in terms of their agonist potency and efficacy to induce MOP receptor-mediated G protein signaling (guanosine-5′-O-(3-[^35^S]thio)-triphosphate ([^35^S]GTPγS) binding) and mobilization of intracellular calcium (calcium fluorescence measurements). The present studies were also conducted to evaluate the antinociceptive activities of the new derivatives **4**–**6** in tests of thermal (hot-plate and tail-flick tests) and chemical (paraphenylquinone (PPQ) abdominal stretching assay) sensitivity. All these investigations may provide further information on key structural features at position 6 in *N*-methylmorphinans that influence affinity, selectivity, efficacy and potency at the MOP receptor.

## Results

### Opioid receptor binding affinity and selectivity

Binding affinities at MOP, DOP and KOP receptors of the new 6-acrylonitrile incorporated *N*-methylmorphinans (**5** and **6**) and the corresponding amido derivative **4** were determined by radioligand binding assays in rat brain membranes. Data as inhibition constant (K_i_) values and selectivity ratios are listed in Table 1. For comparison purposes, opioid binding affinity data for the previously reported 6-cyanomorphinans **1**–**3** and oxycodone are included. Compared to the lead molecule oxycodone, all three new derivatives **4**–**6** displayed a marked increase in MOP receptor affinity. As shown in Table 1, compounds **4**–**6** bound with high affinity at the MOP receptor, with derivative **5** having a K_i_ value of 0.54 nM in the rat brain, paralleled by the highest MOP receptor selectivity. All three compounds had one to two orders of magnitude lower affinities at DOP and KOP receptors. The 6-amido substituted derivative **4** also showed high MOP receptor affinity in the low nanomolar range (K_i_ value of 1.61 nM)), but reduced MOP selectivity. The presence of a 6-cyano group in **5** appears to be favorable for both affinity and selectivity for the MOP receptor, while a 6-amido substitution (**4**) leads to 3 to 9 times lower MOP receptor affinity, and up to 5 and 7 times reduced MOP receptor selectivity vs. DOP and KOP receptors, respectively. In addition, the high affinity at the MOP receptor displayed by the 6-cyanomorphinan **5** in the rat brain was also demonstrated at the recombinant rat MOP receptor expressed in C6 glioma cells (C6_rMOP_, K_i_ = 0.70 nM). In line with findings in the rat brain, low binding affinities were determined for this compound in C6 cells transfected with rat DOP receptors (C6_rDOP_, K_i_ = 56 nM) and Chinese hamster ovary (CHO) expressing human KOP receptors (CHO_hKOP_, K_i_ = 229 nM), thus extending the outcomes on the high MOP selectivity of 5 (80 times vs. DOP and 327 times vs. KOP).

**Table 1:** Binding affinities and selectivities at MOP, DOP and KOP receptors

	**K**_**i**_**(nM)**^**a**^			**Selectivity ratios**	
	**MOP**	**DOP**	**KOP**	**DOP/MOP**	**KOP/MOP**
Oxycodone^b^	43.6 ± 1.5	1,087 ± 246	2,658 ± 367	25	61
14-OMC	35.3 ± 2.1	116 ± 15	454 ± 6	3	13
**1**^**b**^	31.7 ± 2.1	498 ± 79	1,648 ± 201	16	52
**2**^**b**^	2.44 ± 0.13	107 ± 5	364 ± 7	44	149
**3**^**b**^	5.38 ± 0.42	197 ± 29	378 ± 155	37	70
**4**	1.61 ± 0.05	28.8 ± 2.3	105 ± 45	18	65
**5**	0.54 ± 0.04	30.3 ± 2.9	200 ± 40	56	370
**6**	7.39 ± 0.34	239 ± 40	194 ± 68	32	26

^a^Membranes from rat brain were used. ^b^Data from [30]. Values represent the mean ± SEM of at least three experiments each performed in duplicate.

Comparison of the new 6-acrylonitrile 4,5-oxygen bridged **6** to the earlier developed non-bridged analogue **3** depicted no major changes in the MOP affinity and selectivity. It was also noted that methylation of the 4-hydroxy group in compounds **2**, **4** and **5** gives rise to an improved interaction with the MOP receptor. When compared the 14-methoxy and 6-cyano substituted **6** to its 6-keto counterpart 14-*O*-methyloxycodone (14-OMC), it was observed that the presence of a 6-acrylonitrile moiety increases binding to the MOP receptor by about 5 times (Table 1). Also, a similar increase was observed in the case of the other two new 14-methoxy substituted derivatives **4** and **5**. The presence of a hydroxyl group in both 4 and 14 positions (compound **1**) appears to largely affect binding at the MOP receptor. Compared to 6-cyano-*N*-methylmorphinans **1**–**3** described earlier [30], the new analogues **4** and **5** had one order of magnitude lower K_i_ values at the DOP receptor, with the 6-cyano derivative **6** showing comparable and reduced affinity. Also, like the 6-cyanomorphinans **1**–**3**, compounds **4**–**6** retained the decreased binding at the KOP receptor (Table 1).

### Functional activity

**Figure 3:**
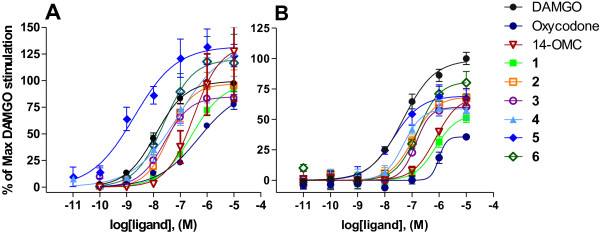
***In vitro *agonist activities at the MOP receptor of compounds 1–6, oxycodone and 14-OMC.** Concentration-response curves in **(A)** [^35^S]GTPγS functional assay with membranes from CHO expressing human MOP receptors and **(B)** calcium mobilization experiments performed with CHO cells co-expressing the human MOP receptor and the Gα_qi5_ protein. Activity is calculated as percentage of maximal DAMGO stimulation in each assay (225 ± 32% and 217 ± 21% in [^35^S]GTPγS functional and calcium mobilization assay, respectively, set as 100%). Calculated potencies and efficacies are presented in Table 2. Data are shown as the mean ± SEM (*n* ≥ 3).

Compounds **1**–**6**, oxycodone and 14-OMC were examined for their agonist potencies and efficacies at the MOP receptor. Stimulation of [^35^S]GTPγS binding and intracellular calcium release were determined and compared to the effect exerted by the reference MOP receptor agonist DAMGO (Table 2). First, we investigated the effects of the *N*-methylmorphinans **1**–**6** at the level of MOP receptor-mediated G protein signaling using agonist-stimulated [^35^S]GTPγS binding in membranes from CHO cells expressing human MOP receptors (CHO_hMOP_). In CHO_hMOP_ cell membranes, all investigated opioid ligands produced concentration-dependent increase in [^35^S]GTPγS binding (Figure 3A). Results showed that the 6-cyano substituted **2**, **3**, **5** and 6, and their amido analogue **4** had one order of magnitude higher potency based on the EC_50_ values than oxycodone and 14-OMC, while the 4,14-dihydroxy-6-cyanomorphinan **1** displayed comparable potency (Table 2). Derivative **5** was the most potent agonist with an EC_50_ value of 1.64 nM, a profile equivalent to the one observed in the radioligand binding studies (Table 1). The new 6-cyanomorphinan **5** proved also to be more potent than DAMGO (EC_50_ = 20.2 nM) in stimulating G protein signaling, while the analogues **1**–**4** showed similar or lower potencies. The 6-amido substituted **4** had an about 16 times higher EC_50_ value compared to its 6-cyano analogue **5**, but similar potency to the other two 6-cyanomorphinans **2**, **3** and **6**. The 4-methoxy analogue **2** had an about 6 times greater potency than its 4-hydroxy counterpart **1**. A further increase in potency resulted upon methylation of the hydroxyl group in position 14, leading to compound **3** with about 10 times lower EC_50_ value than its analogue **1**. The 6-cyanomorphinans **1**–**6** were full agonists, and showed similar efficacies to oxycodone for G protein coupling (Table 2, Figure 3A).

**Table 2:** Agonist activities at the MOP receptor

	**[**^**35**^**S]GTPγS functional assay**^**a**^		**Calcium mobilization assay**^**b**^	
	**EC**_**50**_**(nM)**	**E**_**max**_**(%)**^**c**^	**EC**_**50**_**(nM)**	**E**_**max**_**(%)**^**c**^
Oxycodone	500 ± 128	92 ± 9	1,176 ± 347	38 ± 3
14-OMC	325 ± 94	137 ± 55	973 ± 204	72 ± 7
**1**	273 ± 24	98 ± 4	957 ± 233	57 ± 4
**2**	42.5 ± 14.9	97 ± 15	116 ± 9	69 ± 7
**3**	26.2 ± 1.7	85 ± 3	140 ± 21	61 ± 6
**4**	25.6 ± 9.5	107 ± 26	56.1 ± 11.3	59 ± 8
**5**	1.64 ± 0.19	133 ± 7	21.7 ± 5.5	70 ± 8
**6**	25.1 ± 3.6	121 ± 27	173 ± 21	84 ± 10
DAMGO	20.2 ± 5.6	100	42.7 ± 7.6	100

^a^Membranes from CHO cells stably transfected with human MOP receptors were used. ^b^CHO cells co-expressing Gα_qi5_ protein and recombinant human MOP receptor were used. ^c^E_max_ is expressed in percentage relative to maximal DAMGO induced stimulation in each assay (225 ± 32% and 217 ± 21% in [^35^S]GTPγS functional and calcium mobilization assay, respectively, set as 100%). Values represent the mean ± SEM of at least three experiments each performed in duplicate or triplicate.

We have further assessed the ability of the investigated compounds to activate second messenger systems coupled to the calcium mobilization pathway. Changes in intracellular calcium concentration were determined in CHO_hMOP_ cells expressing the Gα_qi5_ chimeric protein using a whole cell fluorescence-based assay. All compounds evoked a concentration-dependent stimulation of calcium release (Figure 3B). As shown in Table 2, overall the rank order of potencies of compounds **1**–**6** in stimulating calcium release is largely in agreement with the [^35^S]GTPγS findings, with compound **5** being the most potent, followed by the 6-amido analogue **4** (EC_50_ of 21.7 and 56.1 nM, respectively). The other 6-cyano substituted derivatives **1**, **2**, **3** and **6** exhibited up to 10 times greater potency than oxycodone and 14-OMC. Compared to DAMGO (EC_50_ = 42.7 nM), the new 6-cyano substituted **5** was about 2 times more potent, and the other derivatives had EC_50_ values ranging from 56.1 to 957 nM. Generally, the EC_50_ values calculated in the calcium mobilization assays were one order of magnitude higher than those determined in the [^35^S]GTPγS binding assay. Also, some differences in compounds efficacies were noticed between the two assays, where largely lower relative efficacies compared to DAMGO were found regarding intracellular calcium release in CHO_hMOP_ cells. All derivatives **1**–**6** and 14-OMC showed greater efficacies than that of oxycodone (Table 2, Figure 3B).

### Antinociceptive effects

**Figure 4:**
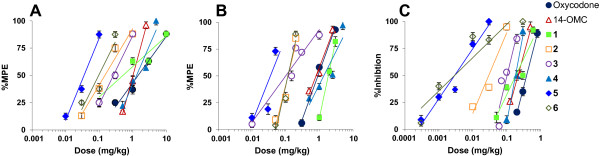
**Dose-dependent antinociceptive effects produced by compounds 1–6, oxycodone and 14-OMC. (A)** Hot-plate test. **(B)** Tail-flick test. **(C)** PPQ abdominal stretching test. Hot-plate and tail-flick latencies (as % MPE) and PPQ-induced stretching response (as % inhibition) were determined as described in Materials and Methods. Data are shown as the mean ± SEM (*n* = 6–10 mice per group).

The new derivatives **4**–**6** were evaluated *in vivo* for their antinociceptive effects in mice after s.c. administration using three well-established and commonly used tests, hot-plate, tail-flick and PPQ abdominal stretching. Both hot-plate and tail-flick assays are valuable models for acute thermal nociception. Activity in the hot-plate test suggests that a drug acts at the supraspinal level, whereas the tail-flick may reflect spinal activity [32]. The PPQ assay evaluates chemical sensitivity, and is established as a model for visceral pain [32]. Dose-dependent antinociceptive effects are illustrated in Figure 4. Antinociceptive potencies expressed as ED_50_ values are listed in Table 3, and were compared with those of the previously reported *N*-methyl-6-cyanomorphinans **1**–**3**[30], oxycodone and 14-OMC.

**Table 3:** Antinociceptive activities

	**ED**_**50**_**(mg/kg, s.c.) (95% CL)**		
	**HP**^**a**^	**TF**^**b**^	**PPQ**^**c**^
Oxycodone^d^	1.37 (0.48-3.92)	0.94 (0.40-2.30)	0.38 (0.19-0.75)
14-OMC	1.02 (0.52-2.01)	0.80 (0.32-2.04)	0.22 (0.12-0.41)
**1**^**d**^	0.50 (0.12-2.02)	1.88 (1.25-2.83)	0.18 (0.076-0.42)
**2**^**d**^	0.15 (0.054-0.41)	0.12 (0.061-0.23)	0.026 (0.012-0.055)
**3**^**d**^	0.25 (0.11-0.59)	0.21 (0.11-0.40)	0.11 (0.072-0.16)
**4**	1.30 (0.56-3.03)	1.34 (0.53-3.03)	0.18 (0.08-0.43)
**5**	0.080 (0.011-0.61)	0.040 (0.020-0.090)	0.0023 (0.0009-0.0060)
**6**	0.089 (0.037-0.21)	0.12 (0.070-0.20)	0.003 (0.0007-0.012)

Antinociceptive potencies in mice after s.c. administration shown as ED_50_ values with 95% confidence limits (95% CL) (*n* = 6–10 mice per group). ^a^Hot-plate (HP) test. ^b^Tail-flick (TF) test. ^c^PPQ abdominal stretching test. ^d^Data from [30].

As shown in Table 3, compounds **4**–**6** produced potent antinociceptive effects in all three *in vivo* assays. The 6-cyano substituted **5** and **6** exhibited markedly higher antinociceptive potencies than the 6-amido analogue **4**, and were up to 165 times more active than oxycodone. The 6-acrylonitrile 4,5-oxygen bridged **6** showed comparable potency to its analogue **5** in the hot-plate and PPQ tests, and it was 3 times less potent in the tail-flick test. Compared to the earlier described derivatives **1**–**3**[30], the new 6-cyanomorphinans **5** and **6** were overall more potent as antinociceptive agents in mice after s.c. administration being highly effective against thermal and chemical nociception. The 6-amido derivative **4** was as potent as compound **1** in the tail-flick and PPQ assays, while it was about 3 times less active in the hot-plate test (Table 3). Antinociceptive potencies of compound **4** were also found to be comparable to those of 14-OMC and oxycodone. The 14-methoxy-6-cyanomorphinan **6** was 11, 7, and 72 times more potent than its 6-keto counterpart 14-OMC in inducing an antinociceptive response in the hot-plate, tail-flick, and PPQ assays, respectively. In addition to antinociception, MOP agonists are well-recognized to elicit other behavioral changes in rodents. In the current study, there were no sedative effects observed at any of the tested doses. Some increase in locomotor activity was noticed in mice, however, this only occurred at doses in the upper end of the dose–response curve, i.e. 90% of analgesia. Further investigations will be needed to establish the side effect profile of these opioids.

## Discussion

Though natural opioid alkaloids such as morphine and codeine (Figure 1) contain a 6-hydroxyl group, synthetic approaches have uncovered that functionalizing position 6 gives rise to a wide range of diverse activities [28]. Thus, position 6 of the morphinan skeleton has been a major target for successful drug developments over the years, leading to various opioid agonists and antagonists that are of importance both for clinical use and research. Oxycodone and oxymorphone (Figure 1), clinically used as opioid analgesics, are two representative examples of structural variation at C-6, where a carbonyl instead of a hydroxyl group is present in position 6. By targeting the chemically highly versatile 6-keto function of morphinan-6-ones as in oxycodone, we have previously reported on a chemically innovative modification giving rise to a novel class of morphinans with acrylonitrile incorporated substructures [29,30]. The resulted acrylonitrile incorporated 4,5-oxygen bridge-opened *N-*methylmorphinans (**1**–**3**, Figure 1) emerged as high affinity and potent MOP antinociceptive agents, with a pharmacological profile comparable to that of their 6-keto counterparts [30]. The interesting approach to incorporate acrylonitrile substructures into morphinans was further explored by our group and resulted in new derivatives [31].

In the present study, combining *in vitro* ligand binding and functional assays and *in vivo* behavioral approaches, we show that the presence of a cyano group in position 6 in *N*-methylmorphinans has a strong influence on opioid receptor binding and post-receptor molecular events. In line with our previous findings, having a 6-cyano group in *N*-methylmorphinans (**5** and **6**) results in increased MOP receptor activity compared to the lead molecule oxycodone both *in vitro* and *in vivo*. In the series of 6-cyanomorphinans, the new derivative **5** was consistently identified to exhibit the highest affinity and selectivity at the MOP receptor and to be the most potent MOP agonist. The design of compound **5** having a 4,14-dimethoxy substitution was attained based on our earlier observations, when a 4-methoxy group and/or a 14-methoxy group, like in compounds **2** and **3**, is more favorable for binding and selectivity for the MOP receptor and antinociceptive activity than the corresponding hydroxy counterpart **1**[30]. Herein, we also establish that the presence of a methoxy group in both positions, 4 and 14, has a major impact not only on binding affinities to all three opioid receptor types, and MOP receptor selectivity, but also on agonist potencies and efficacies at this receptor.

We have also examined how the combination of a C-6 cyano functionality together with a closed 4,5-oxygen bridge (compound **6**) will affect *in vitro* and *in vivo* opioid activities. The two 6-cyanomorphinans **3** and **6** show high and similar affinities at the MOP receptor, and low binding to DOP and KOP receptors. In both functional studies, [^35^S]GTPγS binding and intracellular calcium mobilization, compounds **3** and **6** acted as potent MOP agonists with comparable EC_50_ values, and a somewhat reduced efficacy showed by derivative **3**. *In vivo*, the 6-cyanomorphinan **6** with a closed 4,5-oxygen bridge was more potent than its 4,5-oxygen bridge-opened analogue **3** in inducing an antinociceptive effect in mice after s.c. administration (ca. 3 times in the hot-plate, 2 times in the tail-flick and 37 times in the PPQ tests). Closing of the 4,5-oxygen bridge in the 6-acrylonitrile substituted **3** produces no major changes in interaction with the MOP receptor *in vitro*, but augmented antinociceptive potency. On the other hand, the 14-methoxy-6-cyanomorphinan **6** showed greater MOP receptor affinity and agonist potency than 14-OMC and the 14-hydroxy substituted oxycodone, together with much better antinociceptive properties.

It was of interest to assess the result of the conversion of the 6-acrylonitrile to a 6-amido group on the interaction with opioid receptors, signaling, and the link between antinociceptive efficacy and the mode of action. Since the presence of 4- and 14-methoxy groups was favorable in the case of the 6-cyano substituted *N*-methylmorphinan **5**, the same substitution pattern was applied to the 6-amido analogue **4**. It was remarkable to note that the presence of an amido group in position 6 resulted in high affinity at the MOP receptor and also good MOP selectivity. In the [^35^S]GTPγS functional assay, the 6-amido substituted 4,5-oxygen bridge-opened **4** acted as a highly efficacious agonist at the MOP receptor with several times increased potency than oxycodone, 14-OMC and 4,14-dihydroxy substituted 6-cyanomorphinan **1**. The same profile was depicted for compound **4** when stimulating G protein signaling and intracellular calcium release through MOP receptors. When compared to the 6-cyano analogue **5**, the 6-amido group in **4** appears to largely affect agonist potency, leading to reduced activity, especially in antinociceptive potency. It is possible that differential metabolism of derivatives **4** and **5** may determine the differences in the *in vivo* activity. Primary aliphatic amides are known to be rapidly metabolically hydrolyzed [33], whilst the nitrile group is more stable [34].

In this study, we described the *in vitro* functional activities of the previously reported 6-cyanomorphinans **1**–**3** and oxycodone based on the assessment of MOP receptor-mediated G protein activation and intracellular calcium mobilization. Replacement of the 4-hydroxy group in 6-cyanomorphinan **1** with a 4-methoxy group in analogue **2**, or substitution of 14-hydroxyl in compound **1** with a 14-methoxy group in **3** results in 6 to 10 times enhanced agonist potencies and comparable efficacies, upon the test being used. Compared to the 6-ketomorphinans oxycodone and 14-OMC, the 6-cyano substituted *N*-methylmorphinans **1**–**3** generally displayed higher agonist activity *in vitro*, which correlates well with the *in vivo* results on antinociceptive properties. Among all investigated *N*-methylmorphinans, derivative **5** is the most potent agonist in terms of G protein coupling and changes in intracellular calcium concentration. This MOP agonist potency enhancement of the new 6-cyanomorphinan **5** compared to the other derivatives established in the two functional assays is in agreement with the outcomes from *in vitro* binding assays and nociceptive tests, and supports the importance of the presence of both methoxy groups in positions 4 and 14 in this class of opioid morphinans [30].

The clinically relevant analgesic oxycodone was found as the MOP ligand with the lowest agonist potency in the series of the investigated morphinans. In CHO_hMOP_ cell membranes, oxycodone stimulated [^35^S]GTPγS binding with a EC_50_ value of 500 nM, which is lower than the EC_50_ value of 1.40 μM reported by Thompson et al. in the same cell line [35]. In the same work, a lower relative efficacy as percentage stimulation compared to DAMGO at the human MOP receptor in CHO cells was found for oxycodone (67%), while in our study a higher efficacy, i.e. 92% stimulation relative to DAMGO, was determined (Table 2). Comparable potency (EC_50_ = 373 nM) and lower relative efficacy (66%) for oxycodone to our data was reported in C6_rMOP_ cells [36]. Similarly, in CHO_hMOP_ cells stably expressing the Gα_qi5_ chimeric protein, oxycodone exhibited low activity, by producing stimulation of calcium release with an EC_50_ value of 1,176 nM and an efficacy of 38%. A recent study [37] reported on changes in intracellular calcium levels produced by oxycodone in human embryonic kidney-293 (HEK293) cells co-expressing the human MOP receptor and Gα_qi3_ chimeric protein, with low potency (1.74 μM) and high efficacy (100%). Although 14-OMC also displays low agonist potencies at the human MOP receptor in both functional systems, it shows a similar efficacy compared to oxycodone in [^35^S]GTPγS binding and in calcium mobilization, that is also seen in antinociceptive potency. Mostly, compounds **1**–**6**, oxycodone and 14-OMC were found to be more potent MOP agonists in the terms of G protein activation based on the lower EC_50_ values by one order of magnitude than the EC_50_ values for the calcium signaling, and with lesser efficacies measured in the latter. Presumably these differences may be due to variances in receptor reserve in the two cell lines, and/or possibly membranes vs. intact cells. Differences in signaling may also be regulated by the MOP receptor localization within the plasma membrane [38,39]. Receptor localization within the lipid rafts after agonist binding can promote G protein coupling or recruitment of other intracellular regulatory proteins [40,41]. Over the past years, increased attention has been drawn to the understanding of intracellular signaling pathways that mediate the therapeutic and/or adverse effects of opioid agonists acting at the MOP receptor [42-44]. *In vitro* and *in vivo* studies demonstrate that different opioids can initiate distinct cellular and physiological responses downstream of receptor activation [40,42]. The nature of MOP receptor signaling and regulation are functions not only of the type and structure of the agonist acting at the receptor but also of the cellular environment in which the receptor is expressed [40]. Moreover, the present understanding of MOP receptor function is persistently increasing, as the crystal structure is now available [6].

## Conclusions

In summary, the present study explored *in vitro* and *in vivo* pharmacological activities and signaling of new morphinans substituted in position 6 with acrylonitrile and amido functions as potent agonists and antinociceptives interacting with the MOP receptor. Particularly, the 3,4,14-trimethoxy substituted 6-cyano-*N*-methylmorphinan **5** was identified as the most efficacious MOP agonist of the series, and future studies remain to analyze in more detail pathway-dependent agonist efficacy and signaling, and the side effect profile. Development of novel chemotypes as highly active and selective MOP agonists through targeting position 6 in *N*-methylmorphinans provide important insights into ligand-receptor interaction, and thereby better understanding of the linkage between analgesic efficacy and molecular mode of action. The advances in SAR illustrated in this study serve as a valuable tool for designing molecules with optimal configuration that may aid in identification of opioid therapeutics with more favorable pharmacological features, powerful analgesia and less undesirable effects.

## Materials and methods

### Materials

Opioid radioligands [^3^H][D-Ala^2^,Me-Phe^4^,Gly-ol^5^]enkephalin ([^3^H]DAMGO), [^3^H]5α,7α,8β-(-)*N*-methyl-*N-*[7-(1-pyrrolidinyl)-1-oxaspiro(4,5)dec-8-yl]benzeneacetamide ([^3^H]U69,593) and [^35^S]GTPγS were purchased from PerkinElmer (Boston, USA). [^3^H][Ile^5,6^]deltorphin II was obtained from the Institute of Isotopes Co. Ltd. (Budapest, Hungary). DAMGO, naloxone, tris(hydroxymethyl)aminomethane (Tris), 2-[4-(2-hydroxyethyl)piperazin-1-yl]ethanesulfonic acid (HEPES), unlabeled GTPγS, guanosine diphosphate (GDP) were obtained from Sigma-Aldrich Chemicals (St. Louis, MO, USA). All cell culture media and supplements were from Sigma-Aldrich Chemicals (St. Louis, MO, USA) and Invitrogen (Paisley, UK).

Oxycodone was prepared as described [31]. 14-OMC was prepared according to procedures earlier described [45]. Compounds **1**–**3** were prepared according to the published procedures [29,30]. Derivatives **4**–**6** were synthesized as described [31]. All other chemicals were of analytical grade and obtained from standard commercial sources.

### *In vitro* assays

#### Radioligand binding assays

Binding assays were performed as described previously using rat brain membranes [22], and membranes from C6 glioma cells stably expressing the rat MOP receptor (C6_rMOP_) or the rat DOP receptor (C6_rDOP_), and from CHO cells stably expressing the human KOP receptor (CHO_hKOP_) [36]. Protein concentration was determined by the Bradford method using bovine serum albumin as the standard [46].

Membranes were prepared from Sprague–Dawley rat brains obtained from the Institut für Labortierkunde und Laborgenetik, Medizinische Universität Wien (Himberg, Austria). Binding experiments were performed as in 50 mM Tris–HCl buffer (pH 7.4) in a final volume of 1 ml containing 300–500 μg protein [22]. Rat brain membranes were incubated either with [^3^H]DAMGO (1 nM, 45 min, 35°C), [^3^H][Ile^5,6^]deltorphin II (0.5 nM, 45 min, 35°C) or [^3^H]U69,593 (1 nM, 30 min, 30°C) and different concentrations of the test compound. Nonspecific binding was determined in the presence of 10 μM naloxone. Reactions were terminated by rapid filtration using a Brandel Cell Harvester (Brandel Inc., Gaithersburg, MD) and Whatman GF/B glass fiber filters pre-soaked in 0.1% polyethylenimine for 1 h at 4°C for [^3^H]U69,593, or type GF/C for [^3^H]DAMGO and [^3^H][Ile^5,6^]deltorphin II. Filters were washed three times with 5 ml of ice-cold 50 mM Tris–HCl buffer (pH 7.4) and bound radioactivity was measured by liquid scintillation counting.

C6_rMOP_ cells and C6_rDOP_ cells [47], and CHO_hKOP_ cells [48] were grown to confluence, and used in ligand binding assays. C6 cells were cultured in Dulbecco’s modified Eagle’s medium (DMEM) containing fetal bovine serum (FBS, 10%), under 5% CO_2_ in the presence of geneticin (0.25 mg/ml). CHO cells were maintained in DMEM/Ham F-12 medium containing FBS (10%), under 5% CO_2_ in the presence of geneticin (0.25 mg/ml). Cell membranes (25 μg) were incubated with 0.2 nM [^3^H]diprenorphine and different concentrations of the test ligand in 50 mM Tris–HCl buffer, pH 7.4, for 90 min at 25°C in a final volume of 1 ml. Nonspecific binding was defined with 10 μM naloxone. Samples were filtered through glass fiber filters (45 μm; Schleicher & Schuell, Keene, NH) mounted in a Brandel Cell Harvester (Brandel Inc., Gaithersburg, MD) and rinsed three times with ice-cold 50 mM Tris–HCl buffer pH 7.4. Radioactivity retained on the filters was counted by liquid scintillation counting. All binding experiments were performed in duplicate and repeated at least three times.

#### [^35^S]GTPγS functional assays

CHO cells expressing recombinant human MOP receptors (CHO_hMOP_) were grown in DMEM/Ham F-12 medium supplemented with FBS (10%), penicillin/streptomycin (0.1%), L-glutamine (2 mM) and geneticin (400 μg/ml) [49]. Cell cultures were maintained at 37°C in 5% CO_2_ humidified air. Membranes were prepared in buffer A (20 mM HEPES, 10 mM MgCl_2_ and 100 mM NaCl, pH 7.4) as described [27]. Cell membranes (5 μg) were incubated with 0.05 nM [^35^S]GTPγS, 10 μM GDP and different concentrations of the test compound for 60 min at 25°C, in a total volume of 1 ml. Nonspecific binding was determined using 10 μM GTPγS, and the basal binding was assessed in the absence of test ligand. Samples were filtered over Whatman GF/B glass fiber filters and counted as described for binding assays. All experiments were performed in triplicate and repeated at least three times.

#### Calcium mobilization assays

CHO_hMOP_ stably expressing the C-terminally modified Gα_qi5_ were cultured in DMEM/Ham F-12 containing FBS (10%), penicillin (100 IU/ml), streptomycin (100 mg/ml), L-glutamine (2 mM) geneticin (200 μg/ml) and hygromycin B (100 μg/ml). Cell cultures kept at 37°C in 5% CO_2_ in humidified air were used in the calcium mobilization assays performed as described previously [50]. Cells were seeded at a density of 50,000 cells per well into 96-well black, clear-bottom plates. After 24 h, the cells were loaded with medium supplemented with 2.5 mM probenecid, 3 μM of the calcium sensitive fluorescent dye Fluo-4 AM and 0.01% pluronic acid, for 30 min at 37°C. The loading solution was replaced by Hank’s Balanced Salt Solution (HBSS) supplemented with 20 mM HEPES, 2.5 mM probenecid and 500 μM Brilliant Black, for 10 min at 37°C. After placing both plates (cell culture and compound plate) into the FlexStation II (Molecular Device, Union City, CA), fluorescence changes were recorded. All experiments were performed in duplicate and repeated at least three times.

### *In vivo* assays

#### Animals

ICR male mice (Harlan-Sprague–Dawley, Indianapolis, IN) weighing 20–30 g were used. All procedures involving animals were carried out in accordance to the Guide for the Care and Use of Laboratory Animals, U.S. Department of Health and Human Services, 1985, and were approved by the Institutional Animal Care and Use Committee at Virginia Commonwealth University. Drugs were given by s.c. route. At least three doses were tested, and 6–10 animals per dose were used.

#### Hot-plate test

The hot-plate test was performed in mice using a modified procedure [51] of the earlier described method [52]. Each mouse was exposed to the hot plate (Thermojust Apparatus, Richmond, VA) maintained at 56°C for two trials spaced 5 min apart. Only mice that gave control response latency in the range of 6–10 s on both trials were used. Each mouse received a dose of test drug or vehicle and 30 min later was again tested on the hot plate. Activity was scored as positive if the mouse jumped, licked, or shook its paws at least 5 s beyond its average control latency. Cut-off time was set at 15 s. Percent activity for each dose tested was calculated as (total number of mice scored as positive)/(total number of mice tested) × 100.

#### Tail-flick test

The tail-flick test was performed according to the original procedure described [53] and with some modifications as reported [51]. The mouse’s tail was placed in a groove, which contained a slit under which was located a photoelectric cell. Mice were injected with test drug or vehicle and tested 20 min later. Antinociception was calculated as% MPE (percent maximal possible effect) = (test latency – control latency)/(10 s – control latency) × 100 for each dose tested. A 10 s cut-off was imposed to minimize tissue damage.

#### PPQ abdominal stretching test

The procedure for the PPQ test was described previously [51,54]. Mice were injected with the test drug or vehicle 10 min prior receiving intraperitoneally (i.p.) 2 mg/kg of a freshly prepared PPQ solution. Mice were then placed in three cages in groups of two each. The total number of stretches observed per group during each 1 min period was counted at 10 and 15 min. The total number of stretches for the three groups was determined. A stretch was characterized by an elongation of the mouse’s body, development of tension in the abdominal muscles and extension of the hind limbs. The antinociceptive response was expressed as percentage (%) inhibition of the PPQ-induced stretching response and was calculated as [1 – (total number of stretches in the medicated mice)/(total number of stretches in the control mice)] × 100.

### Data analysis

Binding and functional data were analyzed with the GraphPad Prism software (GraphPad Software Inc., San Diego, CA). Concentration-response curves were constructed and inhibition constant (K_i_, nM), agonist potency (EC_50_, nM) and efficacy (E_max_, as percentage of maximum stimulation with respect to the reference MOP agonist DAMGO) were calculated using nonlinear curve fitting analysis. Data are represented as the mean ± SEM. For *in vivo* assays, the effective dose ED_50_ and 95% confidence limits (95% CL) were calculated using the method of Litchfield and Wilcoxon, 1949 [55].

## Abbreviations

C6_rMOP_ cells: C6 glioma cells stably expressing the rat μ opioid receptor; C6_rDOP_ cells: C6 glioma cells stably expressing the rat δ opioid receptor; CHO cells: Chinese hamster ovary cells; CHO_hDOP_: CHO cells expressing recombinant human δ opioid receptors; CHO_hMOP_: CHO cells expressing recombinant human μ opioid receptors; CHO_hKOP_: CHO cells expressing recombinant human κ opioid receptors; DAMGO: [D-Ala^2^,Me-Phe^4^,Gly-ol^5^]enkephalin; DOP receptor: δ opioid peptide receptor; KOP receptor: κ opioid peptide receptor; MOP receptor: μ opioid peptide receptor; MPE: Maximum possible effect; PPQ: Paraphenylquinone; [^35^S]GTPγS: Guanosine 5′-*O*-(3-[^35^S]thio)triphosphate.

## Competing interests

The authors declare that there are no competing interests.

## Authors’ contributions

Conceived and designed the experiments: MS, HS, GC, JRT, AC. Performed the experiments: TBH, MS, DM, MDA, LSH, JRT. Analyzed the data: TBH, MS, DM, MDA, LSH, JRT, AC. Contributed reagents/materials/analysis tools: MS, HS, GC, JRT, AC. Wrote the paper: TBH, MS, HS. All authors read and approved the final manuscript.
